# Cases of Diseased Bladder and Testicle. Illustrated with Etchings

**Published:** 1816-06

**Authors:** 


*Cases of diseased Bladder and Testicle. Illustrated with Etchings.* By WILLIAM WADD, Surgeon. 4to. pp. 72 London. 1815.

A conviction of the importance of morbid anatomy is at length beginning to be universally diffused, and ardour for its improvement to pervade every class of our profession. That light, which first broke from the sepulchre in the days of Bartholine and Bonetus, now dawns auspiciously on medicine; and the darkness of prejudice, and the dreams of theory, are dissipated in the rising splendour. But there are many parts of the science to which a dubious gleam only has yet penetrated. Much still remains to be discovered; much to be more clearly elucidated; and every attempt directed to these objects, however feeble or imperfect, we hail with pleasure; as it bespeaks a zeal honourable to its author, and must always possess the sterling merit of good intention. Paltry and improper motives can hardly exert their influence where "marvellous cure and wonder-working potion" are not to be blazoned to the world. Zeal alone can animate the pathologist through the drudgery of morbid dissections. The pride of science can be but little gratified by exposing the catalogue of its failures; and professional vanity will rarely choose for the theatre of its exhibitions, a scene hung round with the trophies of the destroyer, and calculated to inspire only a humiliating sense of its own impotency and insignificance.

The work of Mr. Wadd is divided into two parts, respectively entitled, *Diseases of the Bladder and its Appendages;* and *Diseases of the Testicle.* The plates, twenty-one in number, are etched by himself. They are probably his first productions. We shall proceed to consider them individually, and glean from the accompanying histories and explanations every fact that may deserve notice, or at all interest the diagnosis of the morbid affections which they are intended to illustrate.

### DISEASES OF THE BLADDER AND ITS APPENDAGES.

In a sort of introduction to this first part, nothing particularly worthy of remark presents itself. Mr. Wadd reprobates, in a tone somewhat savouring of *causticity,* the indiscriminate employment of the caustic bougie; and insinuates that Sir Everard Home, in his description of the middle lobe of the prostate gland, has mistaken for natural structure what should merely be considered as an enlargement resulting from morbid action.

Plate I, displays two specimens of calculi lodged in the ducts and substance of the prostate. In one of these cases, the gland was large (enlarged?); in the other, of a natural size. Retention of urine is the only symptom mentioned.

Plate II. First Etching — Enlargement of the prostate "with a calculus fixed to the surface." (Was the concretion urinary or prostatic?) Second Etching — Enlargement of the verumontanum. Symptoms in both: Evacuation of "pieces of gravel"; constant irritation at the neck of the bladder, not yielding to ordinary remedies; impracticability of introducing a catheter.

Plate III. Enlarged and ulcerated prostate; the ulceration projecting into the bladder and closing the internal orifice of the urethra. Symptoms: Urine, during the last year of life, constantly tinged with blood; occasional bleeding from the urethra. The etching conveys rather the idea of an ulcerated fungus of the prostate.

Plate IV. Thickening of the bladder, with sacculi of the internal membrane containing calculous concretions. The lateral lobes of the prostate much enlarged; the left slightly ulcerated: a large abscess between the posterior surface of the prostate and the bladder, which had destroyed life. The disease had been of twelve year's date. The symptoms, towards the close, had been pain in the rectum and difficulty in voiding the fæces. The patient for several years, had introduced a catheter himself. This etching is one of the worst and coarsest in the volume. The distances are so ill preserved, that what ought to be a concave, has rather the appearance of a plain or even convex, surface. Several of the ensuing plates exhibit a similar defect.

Plate V. Enlarged prostate and thickened bladder; the surface of the latter covered with coagula. Subject, aged 63; disease of twenty years standing, and much aggravated by the employment of gin. Symptoms: Constant irritation at the neck of the bladder; burning pain in the glans penis; involuntary discharge of urine, and consequent inflammation of the scrotum; pyrexia; headache; rejection of food. After the employment of soothing means, the catheter was with difficulty introduced, and the bladder injected with warm water. Signal relief followed a steady perseverance in this remedy. In two years, the disease recurred with aggravated characters. The bladder could no longer be injected, from impracticability of passing the catheter. Mr. Wadd introduces another case, in illustration of the salutary effects of the *Vesicæ Lotura.* He evidently entertains an opinion of this process far more favourable than that which our own experience hitherto induces us to form respecting it.

Plate vi. Remarkable thickening and contraction of the bladder, consequent on urethral strictures: prostate not enlarged, but unusually firm in texture. Subject, aged 38. No history given. One of the most clear and spirited etchings in the collection.

Plates vii and viii. Specimens of enlarged and ulcerated prostate. The subject of the first case was old, much emaciated, and teased by constant dribbling of urine. The existence of stone had been suspected. By introduction of a finger into the rectum, the morbid size and sensibility of the prostate were discovered. The subject of the second had strictures and fistulæ in perinæo; but the urine passed principally by its natural channel. A sufferer from gout, he was sometimes, during the paroxysm, attacked with strangury and retention of urine; and always relieved by the gum catheter. Two cases are added, to prove the influence of gout in inducing a disordered action of the urinary organs. Our own experience, we think, confirms the observation that, with stricture patients, great irritation of these organs is frequently excited by the use of medicinal springs.

Plate ix. Thickened bladder, with a pouch of the internal membrane lodging calculi: ureters, prostate, and verumontanum, enlarged. The patient had strictures, and, for four years before death, could only void his urine by aid of a catheter. An obstinate retention was with difficulty relieved by that instrument. Had Mr. Wadd paid any attention to arrangement, this plate should have been classed with the fourth. From his opinion that the bladder may be most advantageously punctured through the rectum we cannot but dissent. He recommends that the urine be evacuated by puncturing the urethra where the retention is dependant on stricture situated "not more than two or three inches from the orifice." This brings to our recollection a proposal, recently made by a Birmingham surgeon, to cut down upon the urethra, immediately in front of the prostate, in cases of obstinate retention, and relieve the bladder by the introduction of a female catheter through the gland, or, if that cannot be accomplished, by division of its substance with the scalpel.* Whether this operation merits the high eulogium

* See "Medical and Surgical Remarks." By Edward Grainger, &c.

pronounced upon it by the author in question, future experience must determine. We confess that it wears a rather plausible aspect; and hence recommend it to the notice of our surgical readers.

Plate x. Cancerous fungi covering the nates: the urine voided through fistulous openings in the perinæum, scrotum, and groins.

Plate xi. Four urinary calculi, of various sizes, surrounded, whimsically enough, by outlines of the two largest urinary concretions ever met with in this country; the one, weighing twenty-five ounces, taken from the bladder of Sir Thomas Adams; the other, forty-four ounces, from that of Sir Walter Ogilvie. Both these plates might have been omitted without the slightest detriment to the volume.

Plate xii. Enlarged pelvis of a kidney; the other kidney having been utterly destroyed. In this case, the secretion of urine had never decreased in quantity: and portions of fleshy substance, resembling kidney, had been passed by the urethra: one of the same nature was also found in the bladder.

Diseases of the Testicle, occupy the Second Part of the Work.

Plate xiii. Enlargement of the spermatic process, complicated with hydro-sclerocele and enlargement of the verumontanum. The morbid state of the testis was completely masked by the more urgent symptoms of bladder disease. This forms part of the preparation which furnishes the subject of the lower etching in the second plate.

Plates xiv. xv. and xvi. Specimens of fungus arising from the body of the testicle, and obviously referrible to urethral irritation. The fungus, in the first case, succeeded hernia humoralis, induced by the employment of an astringent injection in severe gonorrhœa. The urethra was strictured, and in an irritable state. The treatment, recommended by the late Mr. Ramsden, had effected a considerable reduction in the size of the testis, without diminishing the fungus, when the patient was suddenly destroyed by pulmonary hæmorrhage. In the second,

---

1815. Mr. Grainger is one of those gentlemen who manœuvre with the scalpel much more expertly than with the goose quill, or, in other words, practice better than they write.

the enlargement of the gland yielded to the same plan; and the fungus was removed by ligature. The third case was complicated with sclerocele. The urethra was affected with strictures. The fungus re-appeared after the application of ligature ; and was finally destroyed by caustic; the morbid condition of the urethra having been previously cured, and the testicle much reduced, by the use of bougies. Mercurial friction completely discussed the remaining testicular enlargement. Thus the experience of Mr. Wadd tends much to confirm the opinions of Mr. Ramsden, and to recommend the practice instituted by that very judicious surgeon in morbid affections of the testicle. These three plates are etched in a light and spirited style.

Nothing new or important arrests our notice in the section entitled *Hydrocele*. Alluding to the assertion of Mr. Ramsden, that obliteration of the cavity of the tunica vaginalis is not essential to the cure of hydrocele, Mr Wadd adds that among his notes

"Is a memorandum which very much confirms Mr Ramsden's opini ›ns. A gentleman underwent the operation in May, 1799. He left town at the end of June. The beginning of July he stated by letter that the hydrocele had returned as large as before the operation ; and in the middle of the next month, he wrote word that it had entirely disappeared. The operation had therefore excited a new action in the parts, and though the effusion of fluid had returned, yet the absorbents had recovered their function."

Adhesion of the tunica vaginalis to the testis does, however, commonly take place after the treatment by injection : but Mr. Wadd thinks that the operation would be improved, if its objects were effected with less irritation, and no change from the original structure of the parts were induced.

"With a view of following Mr Ramsden's plan of curing by only exciting a new action with as little pain as possible, I have so lessened the quantity of wine, that the irritation produced has been such as not to detain the patient at home, after the day on which it was used; and I am inclined to think, that very little irritation of the sacculus is sufficient for the cure of most hydroceles that do not exceed half a pint in the quantity of fluid, nor six months from their first ppearance."

Mr. Wadd considers injudicious the advice given by some surgeons, that the fluid of hydrocele should not be discharged until it has accumulated to the amount of at least a pint. On the contrary, he recommends early evacuation. The absorbents are more likely to act with energy while the original structure of the membranes remains unaltered: and thus also the irritation, resulting from pressure of the fluid on the body of the testis, will be obviated. He concurs with Mr. Ramsden in the opinion that "hydrocele accompanied with a disordered state of the urethra may be cured by bougies alone."

Plates XVII and XVIII. The first displays the tunica vaginalis laid open in common hydrocele. In the latter, are represented two cysts containing fluid, and formed in the cellular structure of the scrotum, exteriorly to the tunica vaginalis. On the supposition that the patient was affected with dropsy, unavailing attempts had been made to cure him by purgatives. This etching is executed with great spirit. We wish that the structure of the cysts had been more minutely described.

Plate XIX. An enormously distended scrotum containing a morbid testicle, and intestine. The urine was discharged from the prepuce, which formed "a sort of navel" on the anterior surface of the immense sac. The subject was aged 52. Four pints of fluid had been evacuated from the hydrocele at two operations. The sac extends nearly as low as the knee-joint.

The last section is upon *Hernia Congenita*. It was the opinion of John Hunter, that the detention of the testicle in the abdomen, after the usual period of descent, is determined by some imperfection of the organ itself: and this explanation Mr. Wadd considers to have been verified by his own experience. In every case of congenital hernia which has fallen under his notice, the gland has invariably been found of "diminutive size and flabby texture." The testicle in its descent to the scrotum after the wonted term, may be mistaken for hernia: hence, on all occasions wherein the existence of rupture is suspected, and particularly in subjects about the fifteenth year, the scrotum should be accurately examined, and the situation of the testis ascertained.

Plates XX and XXI, Represent "an appearance of the testicle in hernia congenita, not unusual," and, in Mr. Wadd's opinion, "not sufficiently noticed." This appearance consists of firm fibrous bands connecting the intestine to the spermatic organ, and formed during the passage of the latter from its original seat in the abdomen.

"The frequent occurrence of these adhesions, explains also the difficulty so frequently met with in reducing such herniæ; and that very difficulty should always remind the surgeon of the probability of such a cause."

Morbid preparations, except when elucidated by a correct and luminous statement of the external phænomena by which the lesion has been characterized, possess an utility extremely limited. It is only "by connecting the sign with the morbid change" that the investigation of diseased structure can be rendered essentially subservient to the interests of practical medicine. With these important truths Mr. Wadd seems not to have been sufficiently impressed, when he entered on the present undertaking. The history attached to each specimen is, in general, brief and defective; nor do we meet with any thing in the former part of the volume, calculated to illustrate the diagnosis of any one of those numerous and obscure maladies which commit such dreadful depredations on the system of the urinary organs.

The plates, though exceedingly unequal in execution, reflect credit on the talent and industry of Mr. Wadd. But the etching needle will never be brought to rival the graver in the representation of morbid structure, where such lightness, and variety, and delicacy of expression are required. Plates of this kind must be correctly and even finely executed, or they are worthless. Economy, on occasions like these should invariably be sacrificed to excellence. *Aut Cæsar aut nullus* is a proposition peculiarly applicable to this subject. Alike deficient in taste and professional ardour is the man who, on examining Dr. Farre's exquisite delineations of morbid liver, or the spirited production with which the talents of Mr. Hodgson have more recently furnished us, would turn aside "to count the cost" at which they are procured.

